# Genetics and Clinical Neuroscience in Intellectual Disability

**DOI:** 10.3390/brainsci12030338

**Published:** 2022-03-02

**Authors:** Corrado Romano

**Affiliations:** 1 Research Unit of Rare Diseases and Neurodevelopmental Disorders, Oasi Research Institute-IRCCS, 94018 Troina, Italy; cromano@oasi.en.it; 2 Section of Medical Biochemistry, Department of Biomedical and Biotechnological Sciences, University of Catania, 95131 Catania, Italy

This editorial summarizes the main information leading to a Special Issue on intellectual disability. The Guest Editor pinpoints the win–win interaction of genetics and clinical neuroscience for the best recognition of new genetic syndromes, including intellectual disability. The use of phenotype-first and genotype-first approaches have shown high efficacy in the above-mentioned task. The Guest Editor has outlined the invaluable usefulness of deep phenotyping in the current times of next generation sequencing.

The American Association on Intellectual and Developmental Disabilities (AAIDD) defines intellectual disability (ID) as “a disability characterized by significant limitations in both intellectual functioning and adaptive behavior as expressed in conceptual, social, and practical skills. This disability originates during the developmental period, which is defined operationally as before the individual attains age 22” [1].

Genetics and clinical neuroscience (CN) are key factors in understanding its complexity.

The impact of genetics on ID was first suspected because of the recurrence of more persons with ID in the same family and the gestalt similarities of some people with ID. The first example paved the way to the study of inherited forms of ID. The X-linked IDs were initially known as a factor of male bias. Subsequently, autosomal recessive IDs were recognized because of the horizontal shape of the pedigree. Lastly, autosomal dominant IDs were discovered after years of undervaluation due to their misclassification as sporadic cases of ID. There was rapid development from the understanding of the pedigrees to the discovery of the candidate genes. The second example relies on syndromic IDs, where ID is only a part of a recognizable syndrome with few or several additional features, leading to a common and unique phenotype. Down syndrome (DS) was certainly the first one to be known, but hundreds more have since been discovered.

CN is the branch of medicine deeply involved in the elucidation of phenotypic issues embedded in neuropsychiatric conditions. As an example, CN discovered the behavioral phenotype of ID syndromes, such as Williams syndrome or DS, the EEG pattern of Fragile X syndrome, the lyssencephalic pattern of the brain cortex in Miller–Dieker syndrome, and so on and so forth.

The reason why genetics and CN are the pillars of the understanding of ID is their reciprocal action. Genetics needs CN for appropriateness and meaningfulness, and vice versa.

However, the way this win–win interaction takes place should be split into at least two terms: before 2006 and after 2006.

The first term has been called the phenotype-first approach. Syndromes such as Down, Cri du Chat, Wolf–Hirschhorn, Williams, Di George, Miller–Dieker, Cockayne, and Cornelia de Lange, to name only a few, were recognized on a list of phenotypic features by eminent clinical geneticists and neuroscientists. For these clinical conditions, the genotype was discovered only afterwards. The strengths of this approach are the sharing of clinical features among expert clinicians. They agree on the fact that a recurrent list of signs makes a single condition. That can be simple when the list is straightforward. Furthermore, this approach does not need a complicated laboratory workup. The weaknesses and shortcomings are the fact that the expert clinicians can sometimes disagree, and the diagnosis can be split between two or more conditions. This can also lead to the belief that the condition does not exist. A further pitfall of such an approach is the uncertainty of the existence of the syndrome up to the time when the genotype is discovered. Several conditions acknowledged in that way were subsequently reconsidered after the discovery of the genotype.

The second term began in 2006, when the first genotype-first syndromes were recognized. The use of new laboratory techniques, such as array-comparative genomic hybridization (arrayCGH), as a first-tier approach in all patients with ID without a peculiar syndromic phenotype led to the so-called genotype-first approach. This meant a first scan for a new pathogenic copy number variant (CNV) in patients with ID, the subsequent collection of patients sharing the same CNV, and eventually the thorough evaluation of the phenotype. For these reasons, such an approach has been also called reverse phenotypics. The first condition discovered in such a way was that associated with the 17q21.31 deletion [2], also called Koolen–de Vries syndrome [3]. Subsequently, a substantial number of new microdeletion/microduplication syndromes have been recognized with the genotype-first approach; 15q13.3 deletion [4], 1q21.1 deletion [5], 16p11.2 deletion or duplication [6], and 16p12.1 deletion [7] syndromes are only a few examples. The progressive implementation of “next generation sequencing” (NGS) techniques, such as Whole Exome Sequencing (WES) and Whole Genome Sequencing (WGS) widened the use of the genotype-first approach to the discovery of new genes associated with ID. *ADNP* [8], *CHD8* [9], *NAA15* [10], *POGZ* [11], *DDX3X* [12] are some examples. The main strength of this approach is the fact that, knowing only a grouping phenotype, such as ID, you can classify new conditions. You do not need to indicate a specific genetic test tailored for a peculiar condition. The main weakness could be the less-careful approach of some clinicians to their patients. They could think: why bother with the phenotype? Let’s perform arrayCGH or WES or WGS, and we’ll find the diagnosis.

In truth, current feasibility studies of genomics and NGS need deep phenotyping and expert CN, only, for example, how do we evaluate a possible Variant of Uncertain Significance (VUS) if we do not check it in patients and look at their respective phenotypes? This has been reinforced, for example, by one article [13] published in the Special Issue “Intellectual Disability: From Genetics to Clinical Neuroscience, and Back”. The authors showed how powerful the genotype-first approach is when used by expert clinicians.

In conclusion, NGS needs next-generation clinicians and deep phenotyping. For this reason, the genotype-first approach does not change the need for close integration of genetics with CN.
